# Hepatitis B and C in Immigrants and Refugees in Central Brazil: Prevalence, Associated Factors, and Immunization

**DOI:** 10.3390/v14071534

**Published:** 2022-07-14

**Authors:** Thaynara Lorrane Silva Martins, Grazielle Rosa da Costa e Silva, Carla de Almeida Silva, Davi Oliveira Gomes, Bruno Vinícius Diniz e Silva, Megmar Aparecida dos Santos Carneiro, Leonora Rezende Pacheco, Natalia Motta de Araujo, Margareth Santos Zanchetta, Sheila Araujo Teles, Karlla Antonieta Amorim Caetano

**Affiliations:** 1 Faculty of Nursing, Federal University of Goias, Goiania 74605-080, Brazil; thaynaralorrane@discente.ufg.br (T.L.S.M.); graziellerosasilva@discente.ufg.br (G.R.d.C.e.S.); carla_silva@discente.ufg.br (C.d.A.S.); davigomes@discente.ufg.br (D.O.G.); leonorapacheco@ufg.br (L.R.P.); sateles@ufg.br (S.A.T.); 2 Institute of Tropical Medicine and Public Health, Federal University of Goias, Goiania 74605-050, Brazil; dinizbruno1410@gmail.com (B.V.D.e.S.); megmar@ufg.br (M.A.d.S.C.); 3 Molecular Virology Laboratory, Oswaldo Cruz Institute, Rio de Janeiro 21045-900, Brazil; natalia.motta.araujo@gmail.com; 4 School of Nursing, Toronto Metropolitan University, Toronto, ON M5B 2K3, Canada; mzanchet@ryerson.ca

**Keywords:** hepatitis, hepatitis B, emigration and immigration, immunization

## Abstract

**Introduction:** Eliminating hepatitis B and C in immigrant and refugee populations is a significant challenge worldwide. Given the lack of information in Brazil, this study aimed to estimate the prevalence of infections caused by hepatitis B and C viruses and factors associated with hepatitis B in immigrants and refugees residing in central Brazil. **Methods:** An observational, cross-sectional, and analytical study was conducted from July 2019 to January 2020 with 365 immigrants and refugees. Hepatitis B was detected by a rapid immunochromatographic test, enzyme immunoassay, and chemiluminescence, and hepatitis C by rapid immunochromatographic test. Multiple analysis was used to assess factors associated with hepatitis B infection. **Results:** Of the participants, 57.8% were from Haiti and 35.6% were from Venezuela. Most had been in Brazil for less than 2 years (71.2%). The prevalence of HBV infection and exposure was 6.6% (95% CI: 4.5–9.6%) and 27.9% (95% CI: 23.6–2.8%), respectively, and 34% had isolated anti-HBs positivity. Reporting a sexually transmitted infection was statistically associated with HBV infection (OR: 7.8; 95% CI: 2.3–26.4). No participant with positive anti-HCV serology was found. **Conclusions:** The study showed that participants were outside the reach of prevention and control actions for hepatitis B. Therefore, public health strategies must be designed to reach, inform, and vaccinate this group.

## 1. Introduction

Hepatitis B and C refer to inflammation of the liver caused by hepatitis B virus (HBV) and hepatitis C virus (HCV), respectively, and are a severe public health problem worldwide [1,2]. Although transmission can occur via parenteral and sexual routes [3], in the epidemiology of hepatitis B, the vertical route is a necessary means of transmission in countries with a high prevalence [4,5].

A modeling study estimated the number of chronic hepatitis B carriers worldwide to be 291,992 million [6]. In addition, current data show that more than 58 million people are infected with HCV [7]. Finally, according to the World Health Organization (WHO), there were three million new hepatitis B and C cases and 1.1 million deaths from these viruses in 2019 [1].

Hepatitis B and C can be acute or chronic and asymptomatic or oligosymptomatic [7,8,9] and have been responsible for the most significant burden of liver cancer. The hepatitis B vaccine has been available since the 1980s and has drastically reduced the prevalence of hepatitis B, changing the epidemiological scenario in countries implementing it [10]. Although no vaccine for hepatitis C exists, it is now considered curable through direct antivirals. However, despite these significant advances, access to hepatitis B vaccination, diagnosis, and treatment of viral hepatitis is a major global challenge.

The World Alliance against Viral Hepatitis and WHO currently promote strategies to eliminate viral hepatitis as a public health problem, to reduce new cases by 90% and deaths from viral hepatitis by 65% by 2030 [11]. On the other hand, to achieve these goals, it is essential to reach socially vulnerable subpopulations at risk of exposure to HBV and HCV [12,13], such as immigrants and refugees, an emerging population worldwide.

Despite not receiving massive influxes of refugees, Brazil is experiencing a gradual increase in the migration of people [14,15,16]. The burden of illness on this group is exacerbated by their shared history (country of origin with high rates of infectious diseases and poor prevention programs) [17,18,19,20] and the difficulties of migration [21,22], in addition to living as individuals on the margins in modern Brazil with an unprepared public sector [23]; these conditions may favor the acquisition and transmission of infectious diseases such as hepatitis B and C [24].

There is little information on living and health conditions, and there appear to be no studies on hepatitis B and C among Brazil’s immigrant and refugee population. Thus, the objective was to estimate the prevalence of viral hepatitis B and C and factors associated with hepatitis B in immigrants and refugees from Goiás, Central-West Brazil.

## 2. Materials and Methods

### 2.1. Study Design

This is an observational, cross-sectional, and analytical study.

### 2.2. Study Location and Target Population

The target population consisted of immigrants and refugees residing in Goiás. Data from the 2015 Migration survey in Goiás show that the State of Goiás has 13,563 residents born in other countries [25]. However, the media shows that immigrant and refugee populations are increasing, mainly among those in vulnerable situations [26]. Thus, the study was performed in this region because it has the highest concentration of immigrants and refugees.

To calculate the sample size, the median HBsAg prevalence (6.1%) presented by Coppola et al. [27] in immigrant populations, a significance level of 95% (α < 0.05), design effect 1.5, and absolute precision of 3% were used. Thus, 367 immigrants were needed to compose the sample. The study included 365 immigrants and refugees.

### 2.3. Inclusion and Exclusion Criteria

The inclusion criteria were to be an immigrant in Goiás and identify with at least one of the following migratory statuses: economic immigrant, refugee, environmentally displaced person, immigrant for humanitarian reasons, immigrant from mixed migratory flows, or stateless person. In addition, individuals who had been in Brazil for more than 10 years and aged less than two years were excluded.

An immigrant is understood to be someone who migrates from one country to another to establish habitual residence. Several reasons can drive this migration, such as economic interests, environmental disasters, studies, medical treatment, conflicts, or persecution. A refugee is an immigrant who left their country due to persecution or another situation that affects human rights and is forced to move to preserve their life or freedom under vulnerable conditions and needs international protection [28,29].

### 2.4. Data Collection

The approach to immigrants and refugees was made with the help of members of non-governmental organizations that serve this population and leaders of immigrant and refugee communities in Goiás. Data collection strategies were defined, and the data collection instrument was constructed in meetings and workshops with established community partners. A convenience sample was proposed due to the lack of knowledge of the current number of immigrants and refugees in Goiás, the difficulty of accessing them, and linguistic and cultural challenges. Study data were collected from July 2019 to January 2020 at the Faculty of Nursing of the Federal University of Goiás and previously arranged community partner sites (churches, migrant pastoral association, Spiritist center).

Data collection instruments were prepared in Portuguese, Spanish, French, and Creole (Haiti). In addition, interviewers fluent in the languages of the immigrants/refugees were trained by the project team.

Initially, the researchers asked eligible participants in the study to read and sign the Free and Informed Consent Form, and those under 18 years of age were consented using the Terms of Informed Consent of Children and Adolescents and Free and Informed Assent. After reading and signing the terms, all participants were interviewed privately, using a structured script containing questions addressing sociodemographic data, immigration, non-sexual characteristics, and sexual characteristics.

Then, 10 mL of blood was collected from each participant, by peripheral venipuncture in the upper arm, for rapid testing of hepatitis B and hepatitis C. After performing rapid testing, the remaining sample (about 9 mL) was poured into test tubes, identified with the number of participants, placed in an air-conditioned thermal box, and sent to the Multi-user Clinical Research Laboratory (LAMPEC) of the Faculty of Nursing/UFG; then, the blood samples were centrifuged to obtain serum and plasma, aliquoted, and stored in a −20 °C freezer until removal for laboratory tests for hepatitis B.

The vaccination coverage estimate was based on hepatitis B serology and vaccination records, either a Brazilian vaccination card or similar from the country of origin.

### 2.5. Serological Tests

At the time of data collection, all blood samples were tested using rapid tests for hepatitis B (rapid immunochromatographic test for qualitative determination of Hepatitis B virus surface antigen; Bioclin-HBsAg commercial kit; sensitivity of 99.9% and specificity of 99.8%) and hepatitis C (rapid immunochromatographic test for qualitative determination of anti-HCV; Abon commercial kit; sensitivity of 99.53% and specificity of 99.78%).

After this step, in the laboratory, all blood samples were retested to detect serological markers of hepatitis B by enzyme immunoassay (ELISA) and chemiluminescence: HBsAg (ELISA, Biokit S.A. Barcelona, Spain, sensitivity 100% and specificity 99.58%); Anti-HBs (ELISA, Biokit S.A. Barcelona, Spain, sensitivity of 99.9% and specificity of 99.4%); Anti-HBc (chemiluminescence, Abbot Core Laboratory, Wiesbaden, Germany, sensitivity of 100% and specificity of 99.90%).

### 2.6. Data Analysis and Processing

Interview data and serological test results were entered into EpiData and analyzed using the Stata 13 statistical package (StataCorp., College Station, TX, USA). Descriptive analysis was performed using frequency distributions, means, and standard deviations. In addition, Chi-squared and Fisher’s exact tests were used to test differences between proportions. Prevalences were calculated with 95% confidence intervals.

Individuals aged 17 years or older were selected to identify the variables associated with HBV infection. This age was selected based on the mean age of first sexual intercourse in the studied group: 16.69 years. Independent variables investigated for the specific outcome with *p* < 0.250 were subjected to multiple logistic regression analyses using the Forward method. Values of *p* < 0.05 were considered significant.

## 3. Results

The sociodemographic characteristics of the participants in this study are shown in Table 1. Most participants were male (57%), between 18 and 50 years old (79.2%; mean of 30 years), single/separated/widowed (53.9%), and reported more than 11 years of education (55.8%; mean of 10 years). In addition, more than 90% of the investigated individuals had some religion (93.1%). Regarding the country of birth, 57.8% were from Haiti.

When asked about their immigration status in Brazil, most responded that they were immigrants (71.8%), and 39.9% had legal permission to stay in Brazil. Regarding their professional situation, 37.3% were unemployed and had lived in Brazil for less than 2 years (71.2%). Regarding the number of people living in the same house, most lived with more than four people (50.1%). Regarding the desire to return to their country, more than half reported yes (51.5%); most participants traveled to Brazil alone (44.2%), and regarding the difficulties faced in Brazil, more than half (61.2%) reported difficulty with the Portuguese language.

Of the total number of immigrants and refugees investigated, 102 had serological markers of exposure to HBV, resulting in an overall prevalence of 27.9% (95% CI: 23.6–32.8%). HBsAg reactivity was found in 24 samples (6.6%; 95% CI: 4.5–9.6%). Isolated anti-HBs, a marker of previous immunization against hepatitis B, was detected in 124 samples (34%; CI: 29.3–39.0).

Table 2 presents data on current or past infection and susceptibility considering the continent of origin. The highest present and past infection rates were found in African immigrants and refugees, followed by Central and South America. In addition, a larger number of people susceptible to HBV were identified among immigrants and refugees from Central America.

After logistic regression, to assess the variables associated with HBV infection, only the variable “history of Sexually Transmitted Infections (STIs)” remained statistically associated with HBsAg (*p* < 0.05) (Table 3).

Prior immunization for hepatitis B was observed in 39.5% (n = 144/365) of the investigated group, either through isolated positivity for the anti-HBs marker (34%; n = 124/365) or a record of three doses of the hepatitis B vaccine (12.6%; n = 46/365). The vaccination card was available for 137 participants, of which 46 had a record of three doses of the hepatitis B vaccine and 91 had one or two doses of the hepatitis B vaccine. Table 4 shows the characteristics of the participants according to serological test for immunization (isolated anti-HBs) or vaccination (three doses of hepatitis B vaccine) status. It can be observed that there was a significant difference between the groups considering gender, their continent of birth, and years living in Brazil (*p* < 0.05). A review of vaccination records revealed that 88.1% started the vaccination schedule in Brazil.

No participant with positive anti-HCV serology was found.

## 4. Discussion

The challenging relationship between immigration and health appears across several dimensions, such as the economy and social integration, and they must be analyzed jointly [30]. Furthermore, most immigrants are brought into a marginal context, with precarious conditions resulting from displacement, no public sector involvement, and insecure working conditions, possibly contributing to illness in this population [23].

Research in Brazil shows that migration is characterized by young adults with an average education level higher than the Brazilian average (8 years of study) [22,31]. For example, according to 2020 OBMigra data concerning immigrants in the Brazilian formal labor market, 44.5% and 21.2% had completed high school and higher education, respectively [32]. The present research also confirmed this profile: the sample was formed predominantly by individuals between 18 and 50 years, and (n = 307) 70.5% reported having at least 10 years of schooling considering those of legal age.

The migration crises in Haiti and Venezuela are particularly relevant to Brazil’s migration policy [33,34]. The present study confirms the predominance of these nationalities; 57.8% of the participants were Haitians and 35.6% Venezuelans.

Regardless of the reasons for immigration, they encounter challenges after arriving in Brazil, and this investigation highlights the language barrier. Of the 356 immigrants who answered the question about the main difficulties in adapting to Brazilian society, more than half (61.2%) spoke Portuguese. This fact may be directly related to other findings in this investigation, such as the high unemployment rate (37.3%). Furthermore, not speaking the national language complicates job opportunities and increases the group’s marginalization [35].

In addition, the language barrier prevents access to health services [23]. Welcoming, diagnosing, treating, and preventing are important axes for the health of immigrants. Many come from regions with high communicable disease rates, especially hepatitis B, and recent or poor childhood vaccination programs in their places of origin, such as Haiti [18,36], Venezuela [37], and Africa [38]. Therefore, it is vital to ascertain any characteristics or life habits of immigrants and refugees residing in Goiás which can contribute to acquiring and maintaining hepatitis B.

In this scenario, the prevalence of HBV infection was 6.6% (95% CI: 4.5%–9.6%), indicating moderate-to-high endemicity. The value found was 18 times higher than the estimated HBsAg prevalence for Brazil’s general population [39]. Furthermore, when comparing the present study’s findings to other rates of HBsAg found in socially vulnerable populations in Brazil, the conclusion is similar. That is, lower prevalences of infection for hepatitis B were found: 2.3% in illicit drug users from the North [40], 0.8% in people living in poverty in the Midwest region [41], and 0.7% in manual sugarcane cutters in the Midwest and Northeast regions [42].

Differences were found when analyzing the prevalence of HBsAg by continent of origin, even compared to other investigations carried out in the countries that mostly comprised the sample, such as Haiti and Venezuela [18,19,43]. In the present study, in Central America (n = 198), where Haitians predominated (89.4%), the frequency of HBsAg was 8.6% (n = 17/198; 95% CI: 5.4–13.3%), while among participants from South America (n = 152), especially Venezuelans (87.2%), it was 3.3% (n = 5/152; 95% CI: 1.4–7.5%). According to the Polaris Observatory, in 2016, Haiti had an estimated 313,000 cases of HBV infection (2.9%; 95% CI: 2.7–4.1%) and Venezuela had about 364,000 cases (1.2 %; CI: 1.1–1.8%) [6].

In the present study, all infection and exposure to HBV cases occurred in individuals over 17 years of age. The variable history of STI (OR: 7.8; 95% CI: 2.3–26.4) was statistically associated with HBsAg positivity. STIs can contribute to acquiring other sexual infections, such as hepatitis B and C, and are related to risk behaviors involving unprotected sex [44,45]. Evaluating the characteristics of the 24 HBsAg positive individuals in our study, 18 reported not having used a condom in the last 12 months, and 12 were married or in a stable relationship.

In Brazil, encouraging condom use is one of Brazil’s central policies for preventing sexually transmitted infections [46]. Indeed, in addition to the previously mentioned behaviors, other social and cultural situations presented by immigrants and refugees can contribute to the inconsistent use of condoms [47,48]. For example, the population was strongly influenced by religion; 93.1% were practitioners of some religion, most of them Protestant, and it is suggested that religion can negatively influence condom use [49]. Finally, it is notable that in Venezuela, in addition to lacking policies for distributing condoms to the population, between 2015 and 2019, a condom cost $199.00, and a box of 36 condoms over $7000.00 [50], so certainly inaccessibility contributes to non-adherence.

Not knowing their serological status among those infected with HBV is noteworthy; of the 24 positive HBsAg, only 2 (8.3%) previously reported being chronic carriers of hepatitis B. This finding may be lower than the estimates for America, where only 18% of hepatitis B cases are diagnosed [51], confirming that Brazilian strategies for early diagnosis in vulnerable groups are not effective [52]; this demonstrates the importance of countries keeping their international commitments to reach the global goals of reducing new infections of viral hepatitis.

In 39.5% (n = 144/365) of the investigated group, protection against hepatitis B was observed, which is below that recommended by the National Immunization Program in Brazil [53]. Indeed, many immigrants and refugees in this study come from regions with recently implemented or poor vaccination programs. This lack of access at home suggests that this percentage of immunized people may have received the vaccine in Brazil. Looking at the vaccination records of the 137 participants who presented a record of any dose of the vaccine against hepatitis, it was possible to identify that 88.1% started their vaccination schedule in Brazil.

Another relevant finding is the large proportion of those immunized or vaccinated from Venezuela with a shorter time living in Brazil. This significant difference suggests discrepancies in how refugees are received, depending on the situation. The forced displacement of thousands of Venezuelans to other countries (especially Brazil) was internationally highlighted, and the Brazilian government was supported by UN agencies and social entities in 2018 to create a reception program for these refugees. Operation Acolhida provided emergency assistance to Venezuelans crossing the border from Rondônia [54], and vaccination against hepatitis B was included. Indeed, the successes of this program must be considered by public administrators and serve as a model of service for immigrants/refugees of other nationalities who enter Brazil.

Concerning hepatitis C, behaviors and life habits may not favor anti-HCV. For example, a low number of blood transfusions, piercing, and tattooing and no cases of illicit drug use characterize a group with reduced risk for acquiring parenterally transmitted diseases, such as hepatitis C [1].

This research has some limitations. First, the convenience sampling used can compromise the external validity. On the other hand, the sociodemographic and migration characteristics presented by the group are in line with the findings of other studies [23,55], suggesting possible representativeness within the investigated nationalities.

## 5. Conclusions

The profile found for hepatitis B among the immigrants and refugees studied was high rate of infection, low knowledge about serological status, presence of sexual risk behaviors, and reduced access to preventive measures, evidenced mainly by the low vaccination coverage for hepatitis B; these findings reinforce that eliminating viral hepatitis in the global context requires targeting specific groups in vulnerable situations.

In the context of immigration, the Global Compact for Migration, addressed by the 2030 Agenda for Sustainable Development, is an essential framework for discussing actions that prevent, diagnose, and treat viral hepatitis. Unfortunately, since 2019, Brazil has not been part of this global agreement. Therefore, the results of this study should present a point of consideration for new public health policies aimed at immigrants and refugees in a national and international context.

The linguistic and cultural barriers were undoubtedly a great challenge for the execution of this work. However, this experience underscored the importance of inclusive health services that establish effective communication within the community.

It is essential to highlight the social impact of this study. All individuals who tested positive for hepatitis B were referred for follow-up and treatment, if necessary, via the existing public free health care system in Brazil (*Sistema Único de Saúde*—SUS).

## Figures and Tables

**Table 1:** Sociodemographic and immigration characteristics of 365 immigrants and refugees from the State of Goiás, Brazil, 2019–2020.

Variables	n = 365	(%)
**Sex**		
Male	208	57.0%
Female	157	43.0%
Age * 30 (12.3) **		
2–11	33	9.0%
12–17	25	6.8%
18–30	135	37.0%
31–50	154	42.2%
≥51	18	5.0%
Education * 10.3 (5.4) ** (NI: 14) ***		
≤10 years	155	44.2%
≥11 years	196	55.8%
Civil Status (NI: 5) ***		
Married/Living Together	166	46.1%
Single/Divorced/Widowed	194	53.9%
Religion (NI: 4) ***		
No	25	6.9%
Yes	336	93.1%
**Country of Birth**		
Brazil (1st Generation Children)	4	1.0%
Colombia	1	0.3%
Cuba	1	0.3%
Equador	1	0.3%
Spain	1	0.3%
Guinea Bissau	15	4.1%
Haiti	211	57.8%
Dominican Republic	1	0.3%
Venezuela	130	35.6%
**Continent of Origin**		
Africa	15	4.2%
Central America	198	54.2%
South America	152	41.6%
**Status in Brazil**		
Immigrant	262	71.8%
Refugee	99	27.1%
Brazilian	4	1.1%
Type of visa to remain in Brazil (NI: 4) ***		
Humanitarian Visa	30	8.3%
Permanent Visa	144	39.9%
Work visa	24	6.6%
Temporary visa	63	17.5%
Student visa	17	4.7%
Refugee	61	16.9%
Application in progress	18	5.0%
Brazilian	4	1.1%
Professional Situation in Brazil (NI: 3) ***		
Permanent contract	120	33.3%
Temporary contract	33	9.1%
Self-Employed	32	8.7%
Occasional Work	9	2.5%
Unemployed	135	37.3%
Student	24	6.6%
Child, not school aged	9	2.5%
Years in Brazil 1.7 (2.2) **		
≤2	260	71.2%
≥3	105	28.8%
Number of people living in the same house 3.3 (2.3) ** (NI: 10) ***		
≤3	177	49.9%
≥4	178	50.1%
Desire to return to their country of origin (NI: 10) ***		
No	121	34.1%
Yes	183	51.5%
Do not Know	51	14.4%
Arrived in Brazil (NI: 10) ***		
Alone	157	44.2%
With Family	156	44.0%
With Friends	42	11.8%
Facing difficulty in Brazil related to (NI: 9) ***		
No difficulty	58	16.3%
Portuguese language	218	61.2%
Finding a job	52	14.6%
Legalization of immigration status	8	2.2%
Employer	1	0.3%
Adapting to work	1	0.3%
Adapting to the climate	7	2.0%
Adapting to food	2	0.6%
Adapting to culture	1	0.3%
Race/ethnicity/color bias	4	1.1%
Problems with health services	3	0.8%
Unstable Conditions	1	0.3%

* Years. ** Mean (standard deviation). *** NI: No Information.

**Table 2:** Data on current infections, past infections, and susceptibility considering the continent of origin of 365 immigrants and refugees from the State of Goiás, Brazil, 2019–2020.

Variable	Present Infection (HBsAg)		Past Infection (Anti-HBs + Anti-HBc or Isolated Anti-HBc)		Susceptible (No Serological Marker for HBV)	
	Positive n = 24 (%)	Negative n = 341 (%)	Positive n = 78 (%)	Negative n = 187 (%)	Yes n = 139 (%)	No n = 226 (%)
**Continent**						
South America	5 (3.3)	147 (96.7)	6 (3.9)	146 (96.1)	57 (37.5)	95 (62.5)
Central America	17 (8.6)	181 (91.4)	62 (31.3)	136 (68.7)	81 (40.9)	117 (59.1)
Africa	2 (3.3)	13 (96.7)	10 (66.7)	5 (33.3)	1 (6.7)	14 (93.3)

**Table 3:** Bivariate and multiple analysis of sociodemographic characteristics, immigration, and factors associated with HBsAg positivity in 311 foreign immigrants from the State of Goiás, 2019–2020.

Variables	Bivariate AnalysisHBsAg (n = 311)				Multiple Analysis ****	
					(n = 299)	
	Total	Positive	Negative	*p*	*p*	OR (95% CI) ***
	n = 311 (%)	n = 24 (%)	n = 287 (%)			
**Sex**						
Female	134 (43.0)	8 (6.0)	126 (94.0)			
Male	177 (57.0)	16 (9.0)	161 (91.0)	*0.315*		
**Age**						
≤30	139 (44.7)	9 (6.5)	130 (93.5)			
≥31	172 (55.3)	15 (8.7)	157 (91.3)	*0.461*		
Education * (NI: 8) **						
≤10 years	112 (37.0)	9 (8.0)	103 (92.0)			
≥11 years	191 (63.0)	15 (7.8)	176 (92.2)	*0.955*		
Civil Status (NI: 5) **						
Sing/e/Divorced/Widow	140 (45.7)	12 (8.6)	128 (91.4)			
Married/Living Together	166 (54.3)	12 (7.2)	154 (92.8)	*0.663*		
Religion (NI: 3) **						
No	18 (5.8)	2 (11.1)	16 (88.9)			
Yes	290 (94.2)	22 (7.6)	268 (92.4)	*0.588*		
**Years in Brazil**						
≤2	211 (67.9)	13 (6.2)	198 (93.8)			
≥3	100 (32.1)	11 (11.0)	89 (89.0)	*0.135*		
**Continent of origin**						
South America	107 (34.4)	5 (4.7)	102 (95.3)			
Central America	189 (60.8)	17 (9.0)	172 (91.0)			
Africa	15 (4.8)	2 (13.3)	13 (86.7)	*0.288*		
Do you have or have you had Hepatitis? ***** (NI: 4) **						
No or do not know	298 (97.1)	22 (7.4)	276 (92.6)			
Yes	9 (2.9)	2 (22.2)	7 (77.8)	*0.150*		
Cases of hepatitis in family or partner? (NI: 4) **						
No or do not know	280 (91.2)	22 (7.9)	258 (92.1)			
Yes	27 (8.8)	2 (7.4)	25 (92.6)	*0.934*		
Blood Transfusion (NI: 4) **						
No or do not know	289 (94.1)	23 (8.0)	266 (92.0)			
Yes	18 (5.9)	1 (5.6)	17 (94.4)	*0.713*		
Alcohol use (NI: 4) **						
No	141 (45.9)	13 (9.2)	128 (90.8)			
Yes	166 (54.1)	11 (6.6)	155 (93.4)	*0.399*		
Non-injection drug use (NI: 4) **						
No	304 (99.0)	24 (7.9)	280 (92.1)			
Yes	3 (1.0)	0 (0.0)	3 (100.0)	*0.612*		
Piercing (NI: 4) **						
No	276 (89.9)	22 (8.0)	254 (92.0)			
Yes	31 (10.1)	2 (6.5)	29 (93.5)	*0.765*		
Surgery (NI: 4) **						
No or do not know	225 (73.3)	17 (7.6)	208 (92.4)			
Yes	82 (26.7)	7 (8.5)	75 (91.5)	*0.777*		
Tattoo (NI: 4) **						
No	284 (92.5)	21 (7.4)	263 (92.6)			
Yes	23 (7.5)	3 (13.0)	20 (87.0)	*0.332*		
Victim of violence (NI: 3) **						
No	256 (83.1)	20 (7.8)	236 (92.2)			
Yes	52 (16.9)	4 (7.7)	48 (92.3)	*0.976*		
Age of first sexual relations * (NI: 32) **						
≤16	136 (48.8)	9 (6.6)	127 (93.4)			
≥17	143 (51.2)	13 (9.1)	130 (90.9)	*0.444*		
History of sexual abuse (NI: 3) **						
No	281 (91.2)	23 (8.2)	258 (91.8)			
Yes	27 (8.8)	1 (3.7)	26 (96.3)	*0.407*		
Sex work (NI: 3) **						
No	299 (97.7)	23 (7.7)	276 (92.3)			
Yes	9 (2.3)	1 (11.1)	8 (88.9)	*0.706*		
Sex under the effects of alcohol or drugs (NI: 3) **						
No	274 (89.0)	20 (7.3)	254 (92.7)			
Yes	34 (11.0)	4 (11.8)	30 (88.2)	*0.360*		
History of Sexually Transmitted Infections (NI: 3) **						
No	293 (95.1)	19 (6.5)	274 (93.5)			1
Yes	15 (4.9)	5 (33.3)	10 (66.7)	*0.000*	0.001	7.8 (2.3 –26.4)
Number of sexual partners in the last 12 months (NI: 12) **						
≤1	250 (83.6)	21 (8.4)	229 (91.6)			
≥2	49 (16.4)	2 (4.1)	47 (95.9)	*0.300*		
Use of a condom with a steady or casual partner in the last 12 months (NI: 6) **						
Yes	102 (33.4)	6 (5.9)	96 (94.1)			
No	203 (66.6)	18 (8.9)	185 (91.1)	*0.361*		
Number of people in the same house (NI: 9) **						
≤3	170 (56.3)	14 (8.2)	156 (91.8)			
≥4	132 (43.7)	9 (6.8)	123 (93.2)	*0.645*		

* Years. ** NI: No information. *** OR: Odds Ratio (95% CI): 95% Confidence Interval. **** Adjusted by years in Brazil and continent of origin. Hosmer–Lemeshow test *p* = 0.2395, ROC curve 0.6921. ***** Variable not included in the logistic regression model because it lacks epidemiological plausibility.

**Table 4:** Characteristics of the participants according to the serological status of immunization (isolated anti-HBs) or vaccination (three doses of hepatitis B vaccine) of 365 immigrants and refugees from the State of Goiás, Brazil, 2019–2020.

Variables	Isolated Anti-HBs or Completely Vaccinated			
	Total n (%)	Positive n = 144 (%)	Negative n = 221 (%)	*p*
Age *				*0.000*
2–17	58	38 (65.5)	20 (34.5)	
18–30	135	52 (38.5)	83 (61.5)	
31–49	145	41 (28.3)	104 (71.7)	
≥ 50	*27*	13 (48.1)	14 (51.9)	
Sex				*0.009*
Male	208	70 (33.7)	138 (66.3)	
Female	157	74 (47.1)	83 (52.9)	
**Continent of Birth**				*0.000*
Central	211	53 (25.1)	158 (74.9)	
South America	138	85 (61.6)	53 (38.4)	
Africa	15	5 (33.3)	10 (66.7)	
Europe	1	1 (100.0)	0 (0.0)	
**Years living in Brazil**				*0.031*
≤1	223	96 (43.0)	127 (57.0)	
2–4	102	38 (37.3)	64 (62.7)	
≥5	40	10 (25.0)	30 (75.0)	
Used Brazilian health services (NI: 6) **				*0.980*
Public	303	119 (39.3)	184 (60.7)	
Private	22	9 (40.9)	13 (59.1)	
No	34	13 (38.2)	21 (61.8)	

* Years. ** No Information.

## Data Availability

Not applicable.
